# Mapping Global Diversity Patterns for Migratory Birds

**DOI:** 10.1371/journal.pone.0070907

**Published:** 2013-08-07

**Authors:** Marius Somveille, Andrea Manica, Stuart H. M. Butchart, Ana S. L. Rodrigues

**Affiliations:** 1 Department of Zoology, University of Cambridge, Cambridge, United Kingdom; 2 Centre d’Ecologie Fonctionnelle et Evolutive, CNRS-CEFE UMR5175, Montpellier, France; 3 BirdLife International, Wellbrook Court, Cambridge, United Kingdom; University of Jyväskylä, Finland

## Abstract

Nearly one in five bird species has separate breeding and overwintering distributions, and the regular migrations of these species cause a substantial seasonal redistribution of avian diversity across the world. However, despite its ecological importance, bird migration has been largely ignored in studies of global avian biodiversity, with few studies having addressed it from a macroecological perspective. Here, we analyse a dataset on the global distribution of the world’s birds in order to examine global spatial patterns in the diversity of migratory species, including: the seasonal variation in overall species diversity due to migration; the contribution of migratory birds to local bird diversity; and the distribution of narrow-range and threatened migratory birds. Our analyses reveal a striking asymmetry between the Northern and Southern hemispheres, evident in all of the patterns investigated. The highest migratory bird diversity was found in the Northern Hemisphere, with high inter-continental turnover in species composition between breeding and non-breeding seasons, and extensive regions (at high latitudes) where migratory birds constitute the majority of the local avifauna. Threatened migratory birds are concentrated mainly in Central and Southern Asia, whereas narrow-range migratory species are mainly found in Central America, the Himalayas and Patagonia. Overall, global patterns in the diversity of migratory birds indicate that bird migration is mainly a Northern Hemisphere phenomenon. The asymmetry between the Northern and Southern hemispheres could not have easily been predicted from the combined results of regional scale studies, highlighting the importance of a global perspective.

## Introduction

Bird migration is a phenomenon that has long fascinated scientists and other observers. An estimated 1,855 bird species (19% of extant species) are migratory, making regular cyclical movements beyond their breeding distribution, with predictable timing and destinations [1]. Much attention has been devoted to bird migration, as exemplified by the nearly 2800 references cited in a recent book on the subject [2], and the 4539 articles in the Web of Science under the topic “bird migration”. This extensive literature has concentrated on aspects such as the behavioural adaptations of migration (e.g. [3]), the evolution of migration (e.g. [4]) and the conservation status of migratory species (e.g. [1]). However, very few studies have investigated bird migration using a macroecological approach.

Macroecology, the study of broad-scale spatial patterns in biodiversity [5], has developed considerably in recent years, and as one of the better-studied taxonomic groups, birds have had a key role in this development. From continental to global scales, bird data have been used to investigate, for example: drivers of species richness patterns [6], [7]; the global distributions of range sizes [8] and body sizes [9], the spatial turnover in species [10], and species extinction risk [11]; and the congruence between richness and endemism [12], [13]. However, despite this extensive body of work, most macroecological studies considered bird species only in their breeding distributions. Very few have addressed one of the most striking features of avian biogeography: the fact that, for a substantial proportion of the species, distributions vary seasonally, and accordingly so do macroecological patterns. The few previous studies that have analysed bird migration from a macroecological perspective (see Appendix S1 for a review), focus mainly on Europe and North America, and often analyse just a subset of the local bird community (e.g. only breeding species; Table S1 in Appendix S1). None are global in scale, and only two span the equator. Nonetheless, these studies reveal some macroecological patterns, in particular an increase in the absolute numbers and in the proportion of migratory species with latitude and increasing climatic seasonality (e.g. [14]–[19]).

We investigated whether these regional patterns can generalise to the global scale by mapping global patterns in the diversity of migratory birds. We used a newly released dataset of digital distribution maps for the world’s birds [20], in which breeding and non-breeding ranges are mapped separately. We mapped global spatial patterns in the seasonal variation in species richness due to migration, the diversity of migratory species, the contribution of migratory birds to local bird diversity, the distribution of narrow-range migratory birds, and of threatened migratory birds. The last two have never (to our knowledge) been investigated, and the first three had only been previously assessed through taxonomically and/or regionally restricted studies. Information on the global distribution of these features of avian communities could shed light on how avian communities are structured, and in particular how they adjust to seasonal environments in high latitudes, with many species vacating during the winter and visiting to exploit the food supply during the summer.

## Materials and Methods

### Spatial Data

Data on the distribution of bird species were derived from BirdLife International and NatureServe (2011) [20], a global dataset compiled as part of the International Union for Nature Conservation (IUCN) Red List assessments for birds [21]. This dataset comprised Geographic Information System (GIS) shapefiles of the distributions of all 9783 extant bird species whose distributions are known. These distribution polygons represent moderately coarse generalizations of species’ distributions derived from the locations of known records, with interpolation by experts (see [22] for further details). Polygons were coded according to species’ presence (1– extant; 2– probably extant; 3– possibly extant; 4– possibly extinct; 5– extinct), origin (1– native; 2– reintroduced; 3– introduced; 4– vagrant; 5– origin uncertain) and seasonality (1– resident; 2– breeding season; 3– non-breeding season; 4– passage; 5– seasonal occurrence uncertain). In this analysis, only polygons coded as presence 1 or 2, origin 1 or 2, and seasonality 1, 2 or 3 were included.

For the purpose of the present study, migratory species were defined as those mapped with at least one polygon coded as breeding or non-breeding (seasonality 2 or 3; some such species also had polygons coded as seasonality 1 for resident populations). Hence, this analysis focuses on species whose annual movements result in predictable, large-scale, changes in bird diversity. It does not cover other forms of migration, such as partial migration (in which only a proportion of a population migrates while the rest remain as residents; [1], [23], fine-scale altitudinal migration (not captured in the maps analysed), differential migration (where migrant individuals comprise just one age-class or sex; [24], and nomadic or irruptive species (in which the movements are not predictable seasonally or geographically; [1], [2]. This study concentrated on geographical patterns over land and therefore marine species were excluded from the analysis (but the terrestrial part of coastal species’ distributions was included).

BirdLife International had also coded, in a separate database, the migratory status of each species. As a cross-validation analysis, we checked whether species classified in this database as “full migrants” (those for which a substantial proportion of the global or a regional population makes regular cyclical movements beyond the breeding distribution, with predictable timing and destinations) matched those that we coded as migratory based on the distribution maps. We resolved any discrepancies (either by improving the maps, or by amending the classification in the database). Our analyses were based on the corrected map dataset (which was incorporated into BirdLife International and NatureServe, 2012; available upon request from the website in reference [25]).

**Figure 1:**
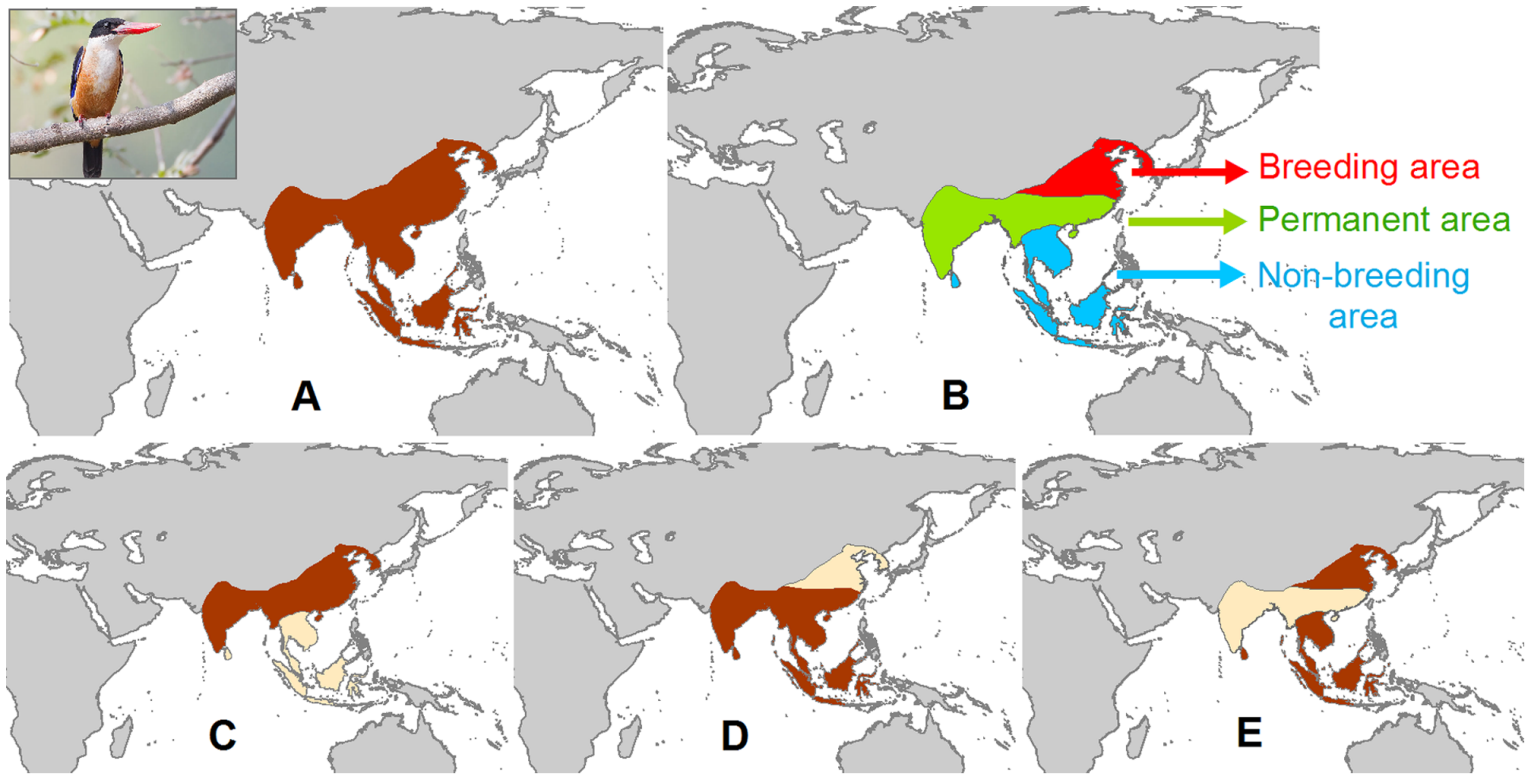
Example of the distribution of a migratory species (Black-capped Kingfisher *Halcyon pileata*). This figure is illustrating: (A) the complete distribution (in brown); (B) subdivision into breeding (red), resident (green) and non-breeding (blue) components; (C) July distribution (including breeding and resident components); (D) January distribution (including non-breeding and resident components); (E) non-permanent distribution (breeding and non-breeding components). Photo by JJ Harrison/Wikimedia Commons.

To investigate the temporal variation between seasons in species distribution, while being globally consistent, we focussed on occupancy in two months, January and July, approximating to the middle of the summer season in the Southern and Northern Hemispheres, respectively. For each species, we assigned occupancy during the two focal months to each component of the distribution (Figure 1). For this purpose, we considered all species whose entire distribution falls within latitudes superior to 30° N as breeding in July and non-breeding in January, and vice-versa for species at latitudes superior to 30° S. For the remaining species, we obtained information on their breeding season from the literature (in particular from the Handbook of the Birds of the World collection; [26]). The few species for which this classification was not applicable were treated for analytical purposes as if they were resident (52 species, mainly tropical). This includes species for which the description of migratory behaviour in the literature was too vague, too conflicting or based on too few observations, as well as species that follow a more complex migration pattern (i.e. partial migrants, differential migrants, nomadic and irruptive species). The non-permanent distribution of each migratory species was defined as the area where the species only occurs seasonally (Figure 1E).

Coastline boundaries were obtained from VMAP, a vector-based collection of GIS data collated by the United States National Geospatial-Intelligence Agency (https://www1.nga.mil/). Antarctica was excluded because no land species occur in this part of the world. The spatial units employed in these analyses were equal-area, equal-shape hexagons, obtained from a geodesic discrete global grid system, defined on an icosahedron and projected to the sphere using the inverse Icosahedral Snyder Equal Area (ISEA) Projection [27]. Hexagons with an area of ∼23,322 km^2^ (corresponding to an ISEA Aperture 3, resolution 7 hexagon grid), were used in all analyses, for a total of 7604 land hexagons.

### Mapping Global Diversity Patterns for Migratory Birds

We produced eight maps to analyse global diversity patterns in birds:

A) Total species richness: mapped as the number of all bird species (migratory and non-migratory) whose distributions intersect with each hexagon. This map does not correspond to the real species richness at any particular time, because migratory species are double-counted in their breeding and non-breeding distributions, but represents the variation in the total species pool across regions.
B) Species richness in January: mapped as the number of bird species (migratory and non-migratory) whose January distributions intersect with each hexagon.
C) Species richness in July: mapped as the number of bird species (migratory and non-migratory) whose July distributions intersect with each hexagon.
D) Difference in species richness: mapped as the number of bird species in July (map B) minus the number of bird species in January (map C) in each hexagon. This map represents the change in species richness between the two opposite seasons due to migration.
E) Total number of non-permanent species (referred hereinafter, for simplicity, as the total number of migratory species): mapped as the number of migratory species whose non-permanent distributions (see Figure 1E) intersect with each hexagon. This represents the spatial variation in the number of species that are only present in each site during part of the year.
F) Fraction of non-permanent species (referred hereinafter as the fraction of migratory species): mapped as the number of migratory species whose non-permanent distributions overlap each hexagon (map E) divided by the total number of species (map A). This represents the fraction of the total species pool that is only present during part of the year.
G) Richness in threatened migratory species: mapped as the number of migratory bird species that are threatened (that is, classified as Vulnerable, Endangered or Critically Endangered according to the IUCN Red List; [21]), per hexagon. As in Map A, species are counted in both their breeding and non-breeding distributions.
H) Richness in narrow-range migratory species. Narrow-range migratory species were defined as those in the lower quartile of migratory species in terms of range size (e.g., [28], and were identified separately for each season (July and January). Separate maps of richness in narrow-range migratory species were then obtained for January and for July, and combined into a final map representing the maximum richness in narrow-range species across seasons.

## Results

**Figure 2:**
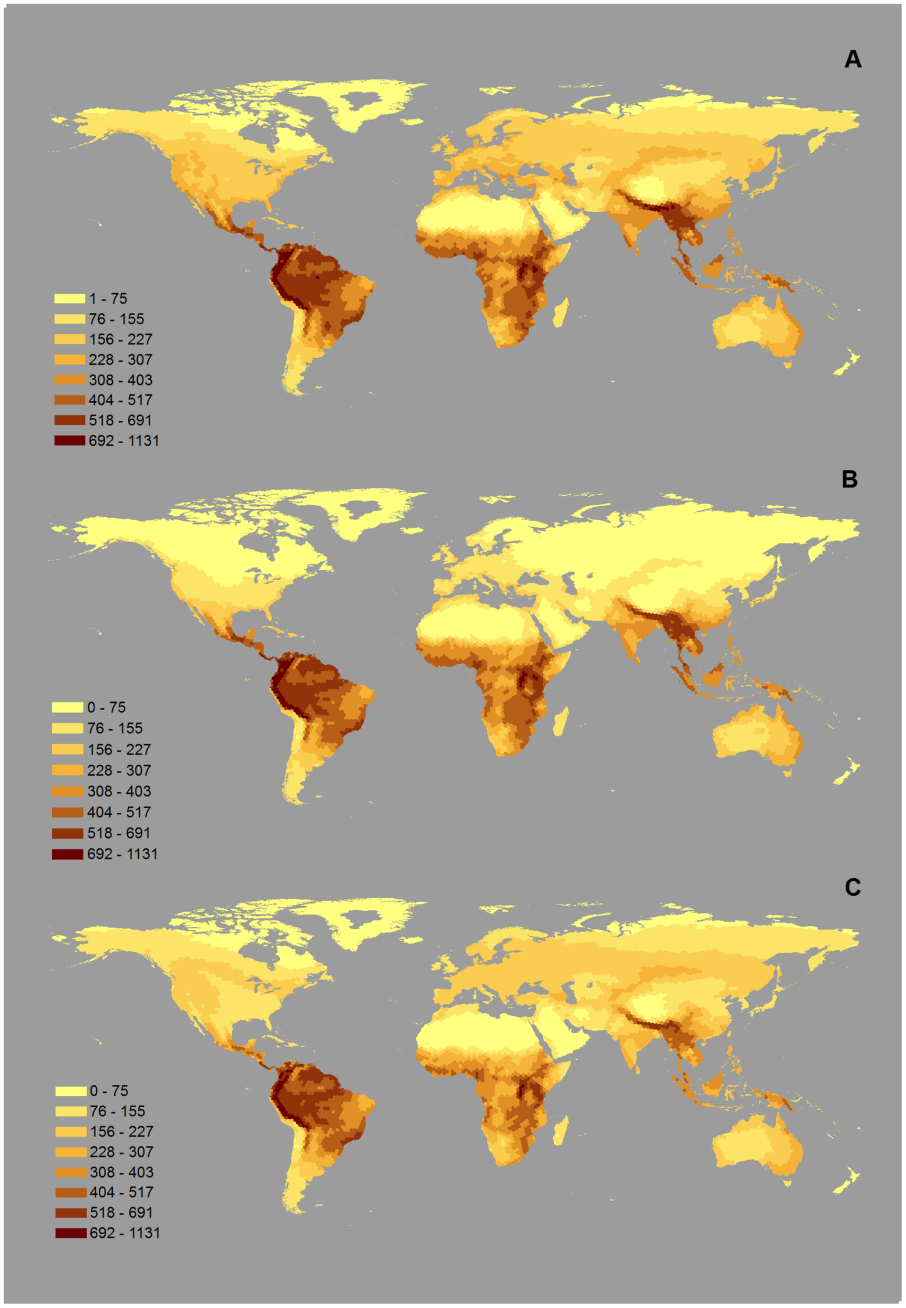
Global patterns of species richness for birds.

**Figure 3:**
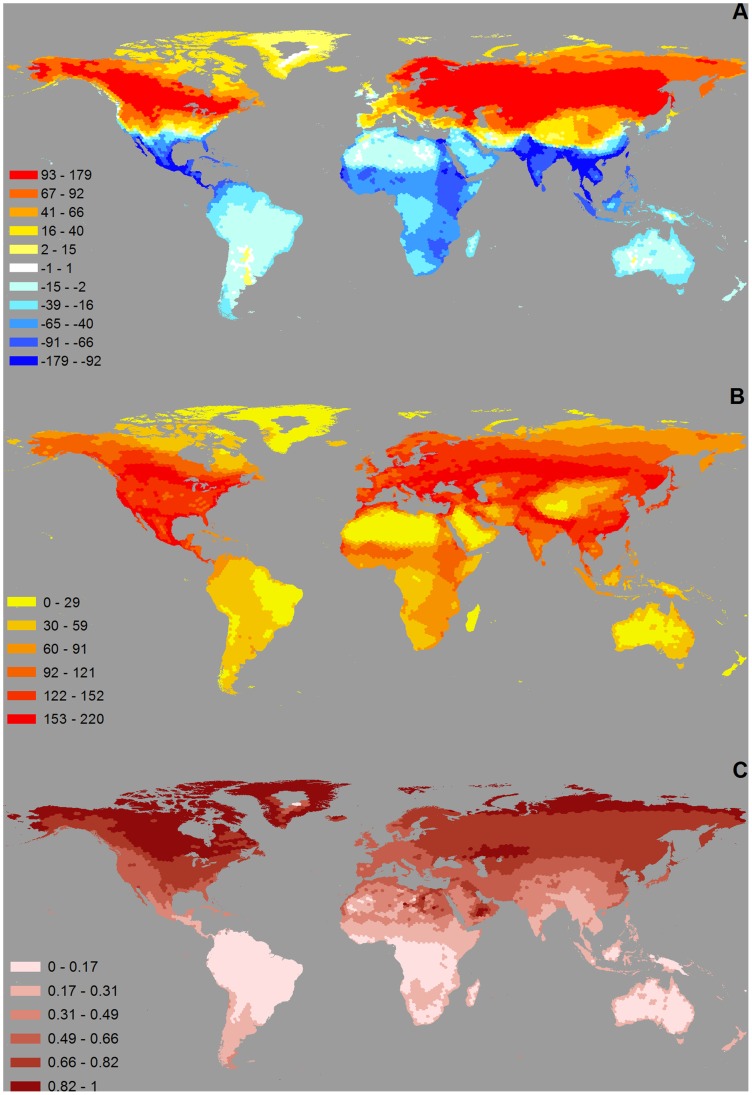
Global patterns for migratory species. (A) Difference in local species richness between July and January, with positive values (in red) indicating areas that are richer in July, and negative values (in blue) indicating areas that are richer in January; (B) richness in migratory species (i.e., non permanent-species, that are only present seasonally in each area); and (C) the proportion of migratory (non-permanent) species.

The global distribution of bird species richness (including both migratory and non-migratory species), is dominated by a concentration of species richness in the tropical regions, especially the tropical Andes in South America, the Eastern Arc mountains of East Africa, and the Himalayan slopes in Asia (Figure 2A). The variation in richness between January (Figure 2B) and July (Figure 2C) is most prominent in the Northern Hemisphere (North America and Eurasia), where species richness is substantially higher in July, but overall this variation is obscured by the magnitude of the latitudinal differences. The seasonal differences become much clearer when mapped directly (Figure 3A). The northern part of the Northern Hemisphere is richer in bird species in July than in January, but in the southern part of the Northern Hemisphere, the reverse is true. A transition zone where the difference in species richness between seasons is small or null (mapped as a pale line in Figure 3A) is found around latitudes 30° to 40° N, crossing North America, the Mediterranean region, Central Asia, the Himalayas and Southern China. In contrast, there is no corresponding transition zone in the Southern Hemisphere. Instead, south of the Northern Hemisphere transition zone, richness is nearly always higher in January, with large seasonal differences in diversity in Central America, South Asia and South-east Asia compared with much smaller seasonal differences in the Sahara, the Arabian Peninsula, South America and Oceania.

Richness in migratory species is considerably higher in the Northern Hemisphere (Figure 3B), with consistently high numbers outside arid regions (Sahara, Arabian Peninsula, Tibetan Plateau) and at high latitudes (Greenland, Northern Russia, Northern Canada). In contrast, richness in migratory species is generally low throughout the Southern Hemisphere. The proportion of migratory species shows a clear latitudinal gradient, increasing towards higher latitudes (Figure 3C). There is, however, a strong asymmetry between the two hemispheres, with higher values in the Northern Hemisphere.

**Figure 4:**
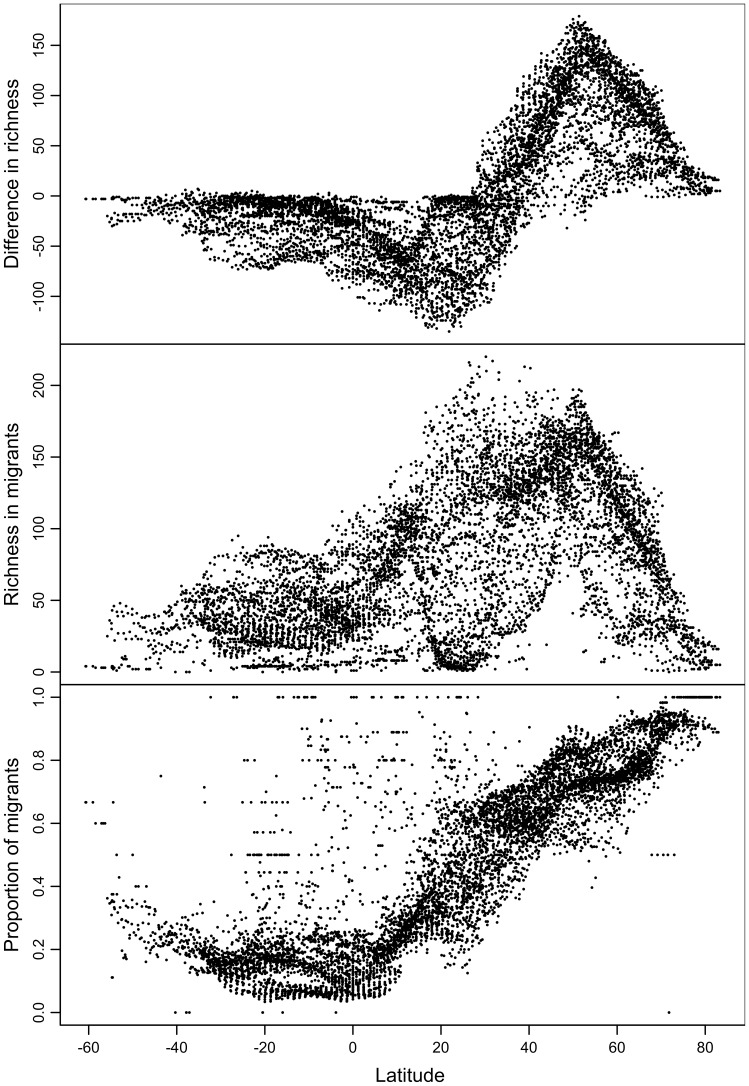
Migratory species diversity as a function of latitude. (A) Difference in local species richness between July and January; (B) richness in migratory (non-permanent) species; and (C) proportion of migratory (non-permanent) species.

The strong asymmetry between hemispheres is most striking when values in the difference in species richness, in the absolute number of migratory species, and in the proportion of migratory species are plotted against latitude (Figure 4). These results are consistent across major migratory flyways (see Appendix S2). The difference in local species richness between January and July is rather uniform (slightly negative) at southern latitudes, whereas at northern latitudes it switches between strongly negative values (<100 species, around latitude 20°N) and strongly positive values (>150 species, around 55°N), with a transition zone around 35°N (Figure 4A). Richness in migratory species is uniformly lower (<100 species) at southern latitudes, whereas in the Northern Hemisphere it reaches up to 200 species between 20 and 55°N, decreasing sharply towards zero at 80°N. Finally, the proportion of migratory species is generally low south of the equator (generally <25%, with a slight increasing trend towards the south), whereas in northern latitudes there is a constant and marked increase from the equator northwards, reaching 100% around 80°N.

**Figure 5:**
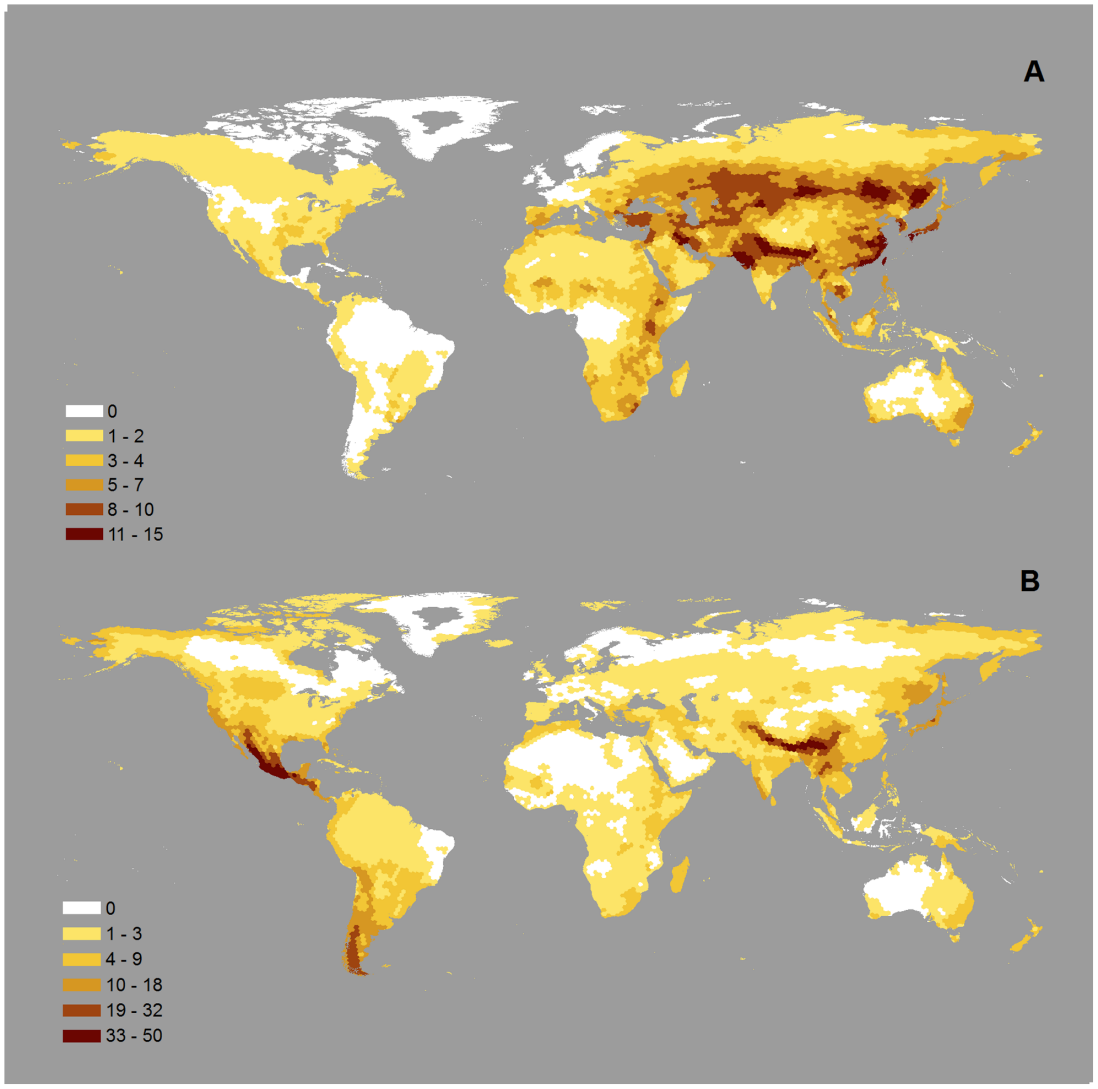
Global diversity patterns for threatened and narrow-range migratory species. (A) Richness in threatened migratory species; (B) richness in narrow-range migratory species.

Among the species we defined as migratory, 132 are globally threatened (Figure 5A). They are mostly concentrated along two parallel bands in Asia, a northern one through Kazakhstan, Mongolia and southern Russia, and a southern band extending from Pakistan, along the Himalayan slopes into southern China and southern Japan, with a smaller concentration in Eastern Africa. Overall, 48% of all threatened migratory species are found in Eurasia, 14% in North America, 12% in South America, 17% in Africa, and 20% in Australasia (the total is larger than 100% because of shared species). Three in every four threatened migratory species also have a narrow range during at least part of the year, particularly those in the Americas and in Australia (see Table S2 in File S2).

The majority (76%) of species classified as narrow-range migratory species was qualified as such in both seasons. The criteria of “narrow” was relative to all migratory bird species, rather than to all birds; in absolute terms, narrow-range migratory species (maximum range size 3.3 million km^2^ in July and 3 million km^2^ in January) have on average substantially wider distributions than narrow-range birds (across all birds “restricted-range species” are defined as those having a breeding distribution size less than 50,000 km^2^ [29]. Richness in narrow-range migratory species is highest in Central America, the Himalayas, and also in Patagonia, and to a lesser extent in South-East Asia and North-East Asia (Figure 5B). In Central America, Patagonia and South-East Asia, these species are found mainly in January, while in the Himalayas and North-East Asia they occur mainly in July (see Figure S3 in File S1).

## Discussion

The results obtained in this study should be interpreted within the context of the limitations in the available data. Species were mapped as polygons that are coarse generalizations of their distribution, and may include relatively extensive areas from which the species is absent, potentially overestimating the species’ true area of occupancy [30]. For some species, finer scale distribution data are available (e.g., [31]), but the data we used represent the best available dataset for all species globally compiled in a consistent manner, thus minimising the degree to which variations in data quality affect the spatial patterns obtained. Even so, distributions maps for species in Europe and North America are likely to be more accurate than those in other regions. However, such limitations are not expected to significantly affect the global patterns obtained, given the coarse spatial resolution of the analyses (cell area c. 23,322 km^2^; [32]. Although lower data quality may result in an underestimation of the total number of birds in the tropics and in the Southern Hemisphere, it would be necessary for hundreds of migratory species to be unmapped for the observed asymmetrical patterns to be offset. This is highly unlikely, as birds are the best known class of organisms (with only 69 species, <1%, classified as Data Deficient, compared with 15% for mammals; [21]). Indeed, lower data quality is likely to minimise rather than exaggerate the observed asymmetry between the northern and southern hemispheres, as coarse distribution maps may overestimate local species richness.

Classification of migratory species was based on the mapped polygons, distinguishing breeding, non-breeding, and resident areas, but this is an oversimplification of the diversity in bird migratory behaviour [33], excluding partial, differential and nomadic or irruptive migrants. Nonetheless, we probably included nearly all species whose annual movements affect the large-scale spatial patterns of diversity of migratory species investigated here. By treating as resident 52 species with complex migratory behaviour or for which there was uncertainty about their distributions (see methods) our estimates of numbers and proportions of migratory species are conservative.

Our results confirm those of Newton and Dale (1997; [34]) in that the difference in species richness between summer and winter is positive in the south and negative in the north of the Western Palearctic region, with a transition around 35°N. Although Newton and Dale only examined species that breed in the Western Palearctic, such species correspond to the vast majority of all migrants in the region (given that the Western Palearctic is the northernmost part of the African-Eurasian flyway, and few species occur as non-breeders in the region while breeding elsewhere). We show that this pattern generalises to the entire Palearctic and Nearctic regions as well, but that there is no corresponding pattern in the Southern Hemisphere (Figure 4A). Moreover, when juxtaposing the spatial pattern in the difference (Figure 3A) with the total number of migratory species (Figure 3B), it becomes clear that the transition area around 35°N (with similar numbers of migratory species in July and January) is an area rich in migratory species, corresponding therefore a region of high seasonal species turnover (in contrast, in areas such as the Sahara, Greenland and central Australia, the small differences between July and January are a consequence of the overall low richness in migratory species). This reveals that the strong migration dynamics in the Northern Hemisphere is dominated by intra-continental (rather than inter-continental) movements, with most species migrating between lower and higher latitudes within the hemisphere. In contrast, the slightly negative values observed in the Southern Hemisphere are mainly due to the relatively few long-distance migrants that cross the equator for the non-breeding season, creating a uniform low enrichment of birds in January. Our results confirm that, globally, bird migration is mainly a Northern Hemisphere phenomenon.

We found a hump-shaped relationship between the absolute number of migratory species and latitude in the Northern Hemisphere, with an increase between the equator and 50°N, followed by a decrease towards the North Pole (Figure 4B). This pattern is very different from that for the distribution of total avian species richness (Figure 2A): bird species in general are concentrated in the tropics, whereas migratory species are concentrated in the northern temperate latitudes. Somewhat unexpectedly, it also differs from the results in previous studies ([18], [35]) which reported a general increase in the absolute number of migratory species with increasing latitude on North America and Europe. The discrepancy may be explained by the fact that these previous studies only looked at the breeding distributions of migratory birds. Indeed, many of the migratory species in the Northern Hemisphere winter in the southern part of this hemisphere, and so high overall numbers of migratory species are also found at relatively low latitudes.

Our study confirms previous results that the proportion of migratory species increases with latitude ([14], [15],b; Figure 3C), and we show that this trend generalises to the global scale. Bird communities at higher latitudes are therefore more influenced by migratory species than those at lower latitudes. However, although this applies to both hemispheres, there is a strong asymmetry in the magnitude of the effect: the local percentage of migratory species reaches a maximum of 60% in the Southern Hemisphere whereas in the Northern Hemisphere percentages are often above this value. This asymmetry is not simply explained by an absence of land at high latitudes in the Southern Hemisphere, as it is found at equivalent latitudes (e.g., about 60% of species at 40°N are migratory, but just 20% at 40°S; Figure 4C). Above 50°N, most communities are composed primarily by migratory species (>50%), which therefore have a major influence on community dynamics (e.g. intra-specific competition [36]) and ecosystem function (e.g., long-distance dispersal of seeds [37] and of pathogens [38]).

Global distribution patterns of threatened and narrow-range migratory species are very different from those for all migratory bird species. The latter are concentrated in the tropical Andes, Atlantic Forests of Brazil, the eastern Himalayas, eastern Madagascar, and the archipelagos of South-East Asia [39] (BirdLife International, 2008) whereas threatened migratory species are concentrated mostly in Asia and to a lesser extent in eastern Africa (Figure 5A). The two latitudinal bands of high diversity of threatened migratory species in Asia are mainly caused by the same species being counted in both their breeding and non-breeding distributions. The local richness in threatened migratory species is much higher in the Eastern Hemisphere than in the Western Hemisphere (Figure 5A), even though the total numbers of threatened migratory species by continent do not reflect this. This is explained by threatened migratory species in the Americas having generally smaller distributions (with lower degree of overlap and so lower richness per hexagon) than in the Eastern Hemisphere.

The majority of threatened migratory species are waterbirds, and these are primarily threatened by widespread degradation and destruction of wetlands [1]. In particular, industrialisation around the Yellow Sea, driven by rapid growth in the population and economy of China and South Korea, affects species on their breeding grounds (e.g., Chinese Egret), non-breeding grounds (e.g., Hooded Crane) or during their migration along the East Asian-Australasian Flyway (e.g., Great Knot, Far Eastern Curlew and Spoon-billed Sandpiper). Furthermore, the intensification of agriculture, combined with the degradation of rivers by pollution and transport in southern Asia, affects many species on their wintering grounds (e.g. Manchurian Reed Warbler, Wood Snipe, Asian Finfoot and Indian Skimmer). The region around the Black and Caspian Seas is another area with many threats to migratory species, such as the intensification of agriculture in Eastern Europe or hunting in Middle East and South-West Asia (e.g. Houbara Bustard; [40]).

When defined across all bird species, narrow-range species are concentrated in the tropics (e.g., northern Andes, Eastern Arc of Africa, and archipelagos of South-East Asia; [13], [29]). In contrast, narrow-range migratory species are mostly absent from those areas and are concentrated in Central America, the Himalayas and Patagonia (Figure 5B). In the Americas, the concentration of migratory species in Patagonia and Central America in January may reflect the more restricted areas of land in these regions, concentrating migrants from the central/northern parts of South America and from North America, respectively. In Asia, the concentration of migrants in the Himalayas in July may reflect the availability of seasonally rich habitats at different altitudes within a narrow latitudinal band, with a large region to the south in South Asia to support migrants in the non-breeding season.

As in previous studies ([13] for birds, [41] for mammals, and [42] for multiple taxa), we found little congruence between areas with high richness, endemism and threat for migratory bird species. The Himalayan region, however, stands out as an exception, suggesting that it is of particular importance for the conservation of migratory birds at the global scale. Other regions highlighted by at least two of these aspects of diversity include south-east Russia, Japan, southern Myanmar, Central America, eastern China, the Rift Valley, north-west India, and Mongolia. Many of these also fall within existing regions of global conservation priority [43]. However, whereas the majority of the latter occur in tropical regions, regions of importance for migratory birds are mainly found in temperate regions in the Northern Hemisphere.

Overall, we found a striking asymmetry between the Northern and Southern Hemispheres in all the patterns investigated (Figures 3, 4 and 5). This was not readily apparent from the combined results of previous regional-scale studies, highlighting the importance of a global perspective for understanding biodiversity patterns. There are a number of (non-mutually exclusive) hypotheses that might explain this asymmetry. First, the evolution of migration from the tropics towards northern areas (as proposed by the “southern-home” theory; [4] might have been favoured by the greater extent of continental mass in the Northern Hemisphere. The shape of the continental mass, with the Southern Hemisphere being dominated by the V-shaped South America and Africa, and isolated islands in Oceania, might have further enhanced this effect. Second, climate seasonality is more extreme in the Northern than in the Southern Hemisphere due to the relative amount of land versus ocean (given the buffering effect of the ocean on climate), making it more challenging for species to remain all year round. Third, differences in climatic history might have provided different opportunities for the evolution of migration in the two hemispheres. Indeed, the Northern Hemisphere has been characterised by much greater long-term climatic variability than the Southern Hemisphere (e.g. more extensive recent glaciations; [44]. Finally, present and past habitat geography could have favoured a more pronounced evolution of migration in the northern hemisphere, for example if important migration routes have evolved between tropical forests and the large temperate forests once occurring in the Northern Hemisphere [45]. Future studies explicitly testing these hypotheses, through their predictions on the spatial patterns of migratory bird diversity, will shed light on the origin and evolution of bird migration.

## Supporting Information

Appendix S1 Literature review. Synthesis of the published literature on bird migration from a macroecological perspective. Information is synthesized in a table (Table S1). (DOC)

Appendix S2 Analysis across flyways. Migratory species diversity as a function of latitude across the three major global migratory flyways. The major global flyways are described in Figure S1. Figure S2 shows the diversity in migratory species plotted against latitude and across flyways. (DOC)

File S1 Figure S3, Global patterns of species diversity for narrow-range migratory species. (A) Richness in narrow-range migratory species in July. (B) Richness in narrow-range migratory species in January. (TIFF)

File S2 Table S2, Summary of geographical location, distribution and major threats for threatened migratory species. (XLS)

## References

[1] Kirby JS, Stattersfield AJ, Butchart SHM, Evans MI, Grimmett RFA, et al (2008) Key conservation issues for migratory land- and waterbird species on the world’s major flyways. Bird Conservation International 18: S49–S73.

[2] Newton I (2008) The Migration Ecology of Birds. 1st ed. London, UK: Academic Press.

[3] Akesson S, Hedenström A (2007) How migrants get there: migratory performance and orientation. BioScience 57: 123–133.

[4] Salewski V, Bruderer B (2007) The evolution of bird migration–a synthesis. Naturwissenschaften 94: 268–279. 17216186 10.1007/s00114-006-0186-y

[5] Gaston KJ, Blackburn TM (2000) Pattern and Process in Macroecology. Oxford, UK: Blackwell Scientific.

[6] Storch D, Davies RG, Zajicek S, Orme CDL, Blackburn TM, et al (2006) Energy, range dynamics and global species richness patterns: reconciling mid-domain effects and environmental determinants of avian diversity. Ecology Letters 9: 1308–1320. 17118005 10.1111/j.1461-0248.2006.00984.x

[7] Davies RG, Orme CDL, Storch D, Owens IPF, Blackburn TM, et al (2007) Topography, energy and the global distribution of bird species richness. Proceedings of the Royal Society B: Biological Sciences 274: 1189–1197. 17311781 10.1098/rspb.2006.0061 PMC2189561

[8] Orme CDL, Davies RG, Olson VA, Thomas GH, Ding T, et al (2006) Global patterns in geographical range size in birds. PLoS Biology 4: 1276–1283. 10.1371/journal.pbio.0040208 PMC1479698 16774453

[9] Olson VA, Davies RG, Orme CDL, Thomas GH, Meiri S, et al (2009) Global biogeography and ecology of body size in birds. Ecology Letters 12: 249–259. 19245587 10.1111/j.1461-0248.2009.01281.x

[10] Gaston KJ, Davies RG, Orme CDL, Olson VA, Thomas GH, et al (2007) Spatial turnover in the global avifauna. Proceedings of the Royal Society B: Biological Sciences 274: 1567–1574. 17472910 10.1098/rspb.2007.0236 PMC2169276

[11] Davies RG, Orme CDL, Olson VA, Thomas GH, Ross SG, et al (2006) Human impacts and the global distribution of extinction risk. Proceedings of the Royal Society B: Biological Sciences 273: 2127–2133. 16901831 10.1098/rspb.2006.3551 PMC1635517

[12] Jetz W, Rahbek C, Colwell RK (2004) The coincidence of rarity and richness and the potential signature of history in centres of endemism. Ecology Letters 7: 1180–1191.

[13] Orme CDL, Davies RG, Burgess M, Eigenbrod F, Pickup N, et al (2005) Global hotspots of species richness are not congruent with endemism or threat. Nature 436: 1016–1020. 16107848 10.1038/nature03850

[14] Newton I, Dale LC (1996) Bird migration at different latitudes in Eastern North America. The Auk 113: 626–635.

[15] Newton I, Dale LC (1996) Relationship between migration and latitude among west European birds. Journal of Animal Ecology 65: 137–146.

[16] Chesser RT (1998) Further perspectives on the breeding distribution of migratory birds ] South American austral migrant ∼ ycatchers. Journal of Animal Ecology 67: 58–66.

[17] Hurlbert AH, Haskell JP (2003) The effect of energy and seasonality on avian species richness and community composition. The American Naturalist 161: 83–97. 10.1086/345459 12650464

[18] Barcena S, Real R, Olivero J, Vargas JM (2004) Latitudinal trends in breeding waterbird species richness in Europe and their environmental correlates. Biodiversity and Conservation 13: 1997–2014.

[19] Carnicer J, Díaz-Delgado R (2008) Geographic differences between functional groups in patterns of bird species richness in North America. Acta Oecologica 33: 253–264.

[20] Birdlife International, NatureServe (2011) Bird species distribution maps of the world. BirdLife International, Cambridge, United Kingdom and NatureServe, Arlington, United States.

[21] IUCN (2012) The IUCN Red List of Threatened Species. Version 2012.1. IUCN. Available: http://www.iucnredlist.org. Accessed 2013 Jul 4.

[22] Buchanan GM, Donald PF, Butchart SHM (2011) Identifying priority areas for conservation: a global assessment for forest-dependent birds. PloS ONE 6(12): e29080. 22205998 10.1371/journal.pone.0029080 PMC3242781

[23] Chan K (2001) Partial migration in Australian landbirds: a review. EMU 101: 281–292.

[24] Jenkins KD, Cristol DA (2002) Evidence of differential migration by sex in White-throated sparrows (Zonotrichia albicollis). The Auk 119: 539–543.

[25] Birdlife International, NatureServe (2012) Bird species distribution maps of the world. BirdLife International, Cambridge, United Kingdom and NatureServe, Arlington, United States. Available: http://www.birdlife.org/datazone/info/spcdownload. Accessed 2013 Jul 4.

[26] Del Hoyo J, Elliott A, Sargatal J (1992–2011) The Handbook of the Birds of the World. Barcelona: Lynx Edicions.

[27] Sahr K, White D, Kimerling AJ (2003) Geodesic discrete global grid systems. Cartography and Geographic Information Science 30: 121–134.

[28] Moore JL, Balmford A, Brooks T, Burgess ND, Hansen LA, et al (2003) Performance of sub-Saharan vertebrates as indicator groups for identifying priority areas for conservation. Conservation Biology 17: 217–218.

[29] Strattersfield AJ, Crosby MJ, Long AJ, Wege DC (1998) Endemic Bird Areas of the World. Priorities for biodiversity conservation. BirdLife Conservation Series 7. Cambridge, UK: BirdLife International.

[30] Hoffman M, Hilton-Taylor C, Angulo A, Böhm M, Brooks TM, et al (2010) The impact of conservation on the status of the world’s vertebrates. Science 330: 1503–1509. 20978281 10.1126/science.1194442

[31] Sauer JR, Hines JE, Fallon JE, Pardieck KL, Ziolkowski Jr DJ, et al.. (2011) The North American Breeding Bird Survey, Results and Analysis 1966–2010. Version 12.07.2011 USGS Patuxent Wildlife Research Center, Laurel, MD.

[32] Hurlbert AH, Jetz W (2007) Species richness, hotspots, and the scale dependence of range maps in ecology and conservation. Proceedings of the National Academy of Sciences USA 104: 13384–13389. 10.1073/pnas.0704469104 PMC1948922 17686977

[33] Dingle H, Drake VA (2007) What is migration? BioScience 57: 113–121.

[34] Newton I, Dale LC (1997) Effects of seasonal migration on the latitudinal distribution of west Palaearctic bird species. Journal of Biogeography 24: 781–789.

[35] Carnicer J, Diaz-Delgado R (2008) Geographic differences between functional groups in patterns of bird species richness in North America. Acta Oecologica 33: 253–264.

[36] Greenberg R, Ortiz JS, Caballero CM (2010) Aggressive competition for critical resources among migratory birds in the Neotropics. Bird Conservation International 4: 115–127.

[37] Whelan CJ, Wenny DG, Marquis RJ (2008) Ecosystem services provided by birds. Annals of the New York Academy of Sciences 1134: 25–60. 18566089 10.1196/annals.1439.003

[38] Reynolds HL, Clay K (2011) Migratory species and ecological processes. Environmental Law 41: 371–391.

[39] BirdLife International (2000) Threatened Birds of the World. Lynx Edicions and BirdLife International, Barcelona and Cambridge.

[40] Tourenq C, Combreau O, Lawrence M, Pole SB, Spalton A, et al (2005) Alarming houbara bustard population trends in Asia. Biological Conservation 121: 1–8.

[41] Ceballos G, Ehrlich PR (2006) Global mammal distributions, biodiversity hotspots, and conservation. Proceedings of the National Academy of Sciences USA 103: 19374–19379. 10.1073/pnas.0609334103 PMC1698439 17164331

[42] Grenyer R, Orme CDL, Jackson SF, Thomas GH, Davies RG, et al (2006) Global distribution and conservation of rare and threatened vertebrates. Nature 444: 93–96. 17080090 10.1038/nature05237

[43] Brooks TM, Mittermeier RA, da Fonseca GAB, Gerlach J, Hoffman M, et al (2006) Global biodiversity conservation priorities. Science 313: 58–61. 16825561 10.1126/science.1127609

[44] Ehlers J, Gibbard PL (2007) The extent and chronology of Cenozoic global glaciation. Quaternary International 164–165: 6–20.

[45] Greenberg R, Kozlenko A, Etterson M, Dietsch T (2008) Patterns of density, diversity, and the distribution of migratory strategies in the Russian boreal forest avifauna. Journal of Biogeography 35: 2049–2060.

