# Supplementary material for: Mapping Global Diversity Patterns for Migratory Birds
Source: PLoS One. 2013 Aug 7;8(8):e70907. doi: 10.1371/journal.pone.0070907 (PMC3737225; doi:10.1371/journal.pone.0070907)
Supplement: Appendix S1 — (DOC) [file pone.0070907.s001.doc]

**Literature Review**

We reviewed the literature on bird migration from a macroecological perspective, by searching for studies that included bird migration in the title, abstract or keywords, covered a regional or global scale (i.e. excluding site-specific or local studies), and involved suites of species. A set of potential papers was first obtained by combining two complementary review strategies: starting from four key papers and searching both within their cited references and within studies that subsequently cited them; and through keyword searches in the Web of Science. The key papers selected as being highly relevant to the present study were Newton and Dale (1996a and 1996b; [1,2]), Herrera (1978; [3]), and Hurlbert and Haskell (2003; [4]). The searches in the Web of Science used the following combinations of key words: “bird*+macroecology”, “migratory bird*+large-scale pattern*”, “bird*+migra*+large spatial pattern*”, “migratory bird*+spatial+pattern*”, “bird migra*+spatial pattern*+global”, “migratory bird*+global pattern*”. The cut-off date for the literature review was 04/09/2011. The potential papers thus obtained were reviewed individually to eliminate those covering local scales or one/few species. Relevant studies were then scrutinised for their geographic location, taxonomic scope, main aim, key findings from a migration perspective, and types of spatial patterns of bird migration investigated (see Table S1).

We found 274 papers that either cited or were cited by the key papers [1–4], whereas the keyword searches in the Web of Science yielded 652 results. Only 27 articles among these (which include the four key papers) fitted the selection criteria (large-scale multi-species migration studies). These included 23 studies that explicitly examined spatial patterns of migratory bird diversity by linking geographic locations and migratory species diversity (see Table S1). The other four studies were excluded from this review: two linked migratory species’ diversity with features other than geographic location (energy availability, [5]; landscape structure, [6]; and two investigated the seasonal variation in range size [7,8].

The selected 23 studies (see Table S1) ranged in geographical extent from national to continental scales, and analysed between fifty-four and over two thousand species of birds. Only four studies focussed on the Southern Hemisphere (three in Sub-Saharan Africa and one in temperate South America), whereas 20 covered the Northern Hemisphere (12 in Europe, six in North America, one in both Europe and North America, and one in Russia). No study was at a global scale, and only two encompassed latitudinal gradients covering both the Northern and the Southern Hemispheres [9,10]. Hockey (2000; [9]) combined Newton and Dale’s (1996a; [1]) European data with results for Sub-Saharan Africa to obtain a large latitudinal span from 35 degrees South to 70 degrees North, and found a symmetrical relationship about the equator between the proportion of residents and latitude. Newton and Dale (1997; [10]), on the other hand, only analysed species that breed in the Western Palearctic, which restricted the coverage south of the equator to long-distant migrants. We found only one study in Asia (covering Russia) and none in Oceania. The majority of studies analysed a subset of the bird species pool occurring in the study area, for example only the breeding species, long-distant migrants, or particular taxa or species groups (e.g. passerines, waterbirds). Six studies analysed the entire avifauna in the study area [1,2,4,9–11], rendering them methodologically closer to our own analysis, but with a more restricted spatial scale (Great Britain, Europe, Eastern North America, Sub-Saharan Africa).

A few general patterns emerge from this review. In Europe and North America, the proportion of migrant species decreases with decreasing seasonality [4,12], and the proportion of breeding migrants increases with latitude, whereas the proportion of non-breeding (wintering) migrants decreases [1,2]. The absolute number of breeding migrant species increases with latitude [13,14]; the number and proportion of migrants increases with longitude (from West to East) in Europe [15,16]; and species diversity in the Western Palearctic is higher in summer in the north and higher in winter in the south [10]. In both Sub-Saharan Africa and Europe, the proportion of migrant species was well predicted by the average temperature of the coldest month [9,16]. The number of wintering long-distance migrant species in Sub-Saharan Africa was found to decrease with increasing latitude [17,18], and in temperate South America the proportion of austral migrants was found to increase with latitude [19]. Some studies have quantified numbers of individuals rather than species diversity, finding that in the boreal forests of Russia, the local abundance of individuals of both short- and long-distance migrant species is correlated with both climate and longitude [20], and that in Europe and North America the local abundance of migrant birds showed a concave curve when plotted against latitude, while the proportion of all individuals that are migrants generally increases with latitude [21].

References

1. Newton I, Dale LC (1996) Relationship between migration and latitude among west European birds. Journal of Animal Ecology 65: 137–146.

2. Newton I, Dale LC (1996) Bird migration at different latitudes in Eastern North America. The Auk 113: 626–635.

3. Herrera CM (1978) On the breeding distribution pattern of European migrant birds: Macarthur’s theme reexamined. The Auk 95: 496–509.

4. Hurlbert AH, Haskell JP (2003) The effect of energy and seasonality on avian species richness and community composition. The American Naturalist 161: 83–97.

5. Monkkonen M, Forsman JT, Bokma F (2006) Energy availability, abundance, energy-use and species richness in forest bird communities: a test of the species-energy theory. Global Ecology and Biogeography 15: 290–302.

6. Flather CH, Sauer JR (1996) Using landscape ecology to test hypotheses about large-scale abundance patterns in migratory birds. Ecology 77: 28–35.

7. Mills AM (2006) Winter range compression of migrants in Central America. Journal of Avian Biology 37: 41–51.

8. Henningsson SS, Alerstam T (2008) Does migration promote or restrict circumpolar breeding ranges of arctic birds? Journal of Biogeography 35: 781–790.

9. Hockey PAR (2000) Patterns and correlates of bird migration in Sub-Saharan Africa. EMU 100: 401–417.

10. Newton I, Dale LC (1997) Effects of seasonal migration on the latitudinal distribution of west Palaearctic bird species. Journal of Biogeography 24: 781–789.

11. Lennon JJ, Greenwood JJD, Turner JRG (2000) Bird diversity and environmental gradients in Britain: a test of the species-energy hypothesis. Journal of Animal Ecology 69: 581–598.

12. Bohning-Gaese K, Lemoine N (2004) Importance of Climate Change for the ranges, communities and conservation of birds. Advances in Ecological Research 35: 211–236.

13. Carnicer J, Diaz-Delgado R (2008) Geographic differences between functional groups in patterns of bird species richness in North America. Acta Oecologica 33: 253–264.

14. Barcena S, Real R, Olivero J, Vargas JM (2004) Latitudinal trends in breeding waterbird species richness in Europe and their environmental correlates. Biodiversity and Conservation 13: 1997–2014.

15. Lemoine N, Bohning-Gaese K (2003) Potential impact of global Climate Change on species richness of long-distance migrants. Conservation Biology 17: 577–586.

16. Schaefer H, Jetz W, Bohning-Gaese K (2008) Impact of climate change on migratory birds: community reassembly versus adaptation. Global Ecology and Biogeography 17: 38–49.

17. Newton I (1995) Relationship between breeding and wintering ranges in Palaearctic-African migrants. Ibis 137: 241–249.

18. Wisz MS, Walther BA, Rahbek C (2007) Using potential distributions to explore determinants of Western Palaearctic migratory songbird species richness in sub-Saharan Africa. Journal of Biogeography 34: 828–841.

19. Chesser RT (1998) Further perspectives on the breeding distribution of migratory birds: South American austral migrant flycatchers. Journal of Animal Ecology 67: 69–77.

20. Greenberg R, Kozlenko A, Etterson M, Dietsch T (2008) Patterns of density, diversity, and the distribution of migratory strategies in the Russian boreal forest avifauna. Journal of Biogeography 35: 2049–2060.

21. Monkkonen M, Forsman JT (2005) Ecological and biogeographical aspects of the distribution of migrants versus residents in European and North American forest bird communities. In: Greenberg R, Marra PP, editors. Baltimore: The Johns Hopkins University Press. pp. 131–142.

**Table S1:** Synthesis of published macroecological studies on migratory birds.

| **Ref.** | **Geographic location** | **Taxonomic Scope** | **Is the main aim about migration?** | **Key findings from a bird migration perspective** | **Diff^a^** | **Rich^b^** | **Prop^c^** |
| --- | --- | --- | --- | --- | --- | --- | --- |
| 1 | Europe (10 million km2) | 154 species (only breeding waterbirds) | No | Southward increase in the number of resident species and southward decrease in the number of aestival species. Seasonality, excess of energy, and competition with residents suggested to affect the distribution of breeding visitors. | No | Yes* | No |
| 2 | Europe (10 million km2) | 300 species (only breeding species) | No | Climate change (particularly the increase in minimum temperatures and decrease in seasonality) is suggested to result in a decline in the proportion of migratory individuals and species within communities. | No | No | Yes* |
| 3 | North America (20 million km2) | ? (only breeding birds) | No | Increase of migratory species richness, and decrease of resident species richness, with latitude. Migratory species were dominant in northern areas while residents were dominant in southern areas. June NDVI was the only good predictor of migratory species richness. | No | Yes* | No |
| 4 | Temperate South America (7 million km2) | 74 species (only Flycatchers) | Yes (breeding distribution of migratory birds) | Strong associations of latitude and temperature with percentage of migrants. | No | No | Yes* |
| 5 | Britain and Ireland (300,000 km2) | 54 species (only passerines) | Yes (geographical and habitat distribution of migrants) | Migrants were less widespread than residents. Residents used a greater diversity of nesting habitats than migrants. Density of migrants and ratio of migrants to residents differed greatly between habitat types. | No | Yes | No |
| 6 | Boreal forest in Russia and Canada (12 million km2) | Russia: 139 spp; Canada: 68 spp (only breeding birds; nocturnal, waterfawls, shorebirds excluded) | Yes (distribution of migratory strategies) | The number of tropical migrants increased along a gradient from west to east, whereas the number of short-distance migrants increased from east to west. The distribution patterns for migrant types were related to both climate and geographical location. | No | Yes | Yes |
| 7 | Western/northern Europe (5 million km2) | ? (only breeding long-distance migrant insectivorous passerines) | Yes (geographical and habitat distribution of migrants) | Clear latitudinal gradient (and to a lesser extent longitudinal gradient) in the proportion of migrants in Europe, with the highest proportion in Northern and Central Europe. The proportion of migrants was consistently low in the tallest vegetation. | No | No | Yes |
| 8 | Western Europe (5 million km2) | ? (only passerine breeding birds) | Yes (breeding distribution of migratory birds) | Latitude was the most important factor in determining the relative importance of tropical migrants in bird communities and this relation continued to hold when the different habitat types are considered individually. | No | No | Yes* |
| 9 | Sub-Saharan Africa (20 million km2) | ~2000 species? (only breeding intra-African and Palearctic migrants) | Yes (patterns and correlates of bird migrations) | The proportion of species that are migratory could be predicted with considerable accuracy from the average temperature of the coldest month of the year. | No | Yes | Yes |
| 10 | North America (20 million km2) | ? (all birds) | No (but investigates avian community composition) | The number and proportion of migrant species in breeding communities increased with the degree of seasonality. The seasonality in NDVI was a good predictor of how many species invade the community during the breeding season. | No | Yes* (winter vs. summer) | Yes |
| 11 | Europe (10 million km2) | 300 species (only breeding species) | Yes (impact of Climate Change on long-distant migrants) | Number and proportion of long-distance migratory species increases with increasing spring temperature and decreasing temperature of the coldest month. Bird communities dominated by short-distance migrants in W Europe, and long-distance migrants in E Europe. | No | Yes | Yes |
| 12 | Britain (243,000 km2) | 150-250 species (all landbirds) | No | Output: maps of diversity in summer visitors or in winter visitors. Winter diversity better predicted from environmental factors than from summer diversity. | No | Yes* (winter vs summer) | No |
| 13 | North America (20 million km2) | ? (only breeding birds) | Yes (breeding distribution of migratory birds) | No simple correlation found between climate and the proportion of migrants. The proportion of neo-tropical migrants was highest in the deciduous forest of the northeast, and negatively correlated with the amount of conifers, grass and general aridity. | No | No | Yes* |
| 14 | Europe + North America (30 million km2) | Europe: 106 spp; N America: 98 spp (only forests birds) | Yes (distribution of migrants versus residents) | Proportion of short-distance migrants increased with latitude. Proportion of long-distance migrants increased with latitude in Europe and was concave in North America. | No | Yes | Yes |
| 15 | Sub-Saharan Africa (20 million km2) | 187 species (only wintering Palearctic migrants) | Yes (breeding vs wintering ranges relationship) | The sizes of breeding and wintering ranges were correlated, but in most species wintering ranges were smaller than breeding ranges. Decline in migrant species numbers from the north of the African wintering range southwards. | No | Yes* | No |
| 16 | Western Europe (5 million km2) | 418 species (all birds) | Yes (migration vs latitude) | Proportion of species that migrate south for winter increased with latitude, and proportion of species that migrate north for summer decreased with latitude. The latitudinal trend in species numbers was more marked in winter. Above 35°N summer species outnumber winter ones. | No | No | Yes* |
| 17 | Eastern North America (10 million km2) | 374 species (all birds) | Yes (relationship between migration and latitude) | Proportion of species that move south for the winter increased with latitude, and proportion of species migrating north for the summer decreased with latitude. The latitudinal trend in species numbers was more marked in winter than in summer. Above 35°N summer species outnumber winter ones, and below 35°N the reverse holds. | Yes | No | Yes* |
| 18 | Europe and Africa (40 million km2) | 488 species (only breeding species) | Yes (effect of migration on latitudinal distribution of bird species) | Highlighted the great seasonal changes in avifaunal distribution that result from regular migration, and the extensive latitudinal shifts involved. The latitudinal spans of breeding and wintering ranges were correlated. Species richness is higher in summer than winter above 35°N and vice-versa below 35°N. | Yes (winter vs summer) | No | No |
| 19 | Europe (10 million km2) | 161 species (only waterbirds) | No (but investigates avian community composition) | Low energy availability of the northern basins in the wintering season proposed as a limit to the distribution of many wintering species, whereas the higher energy level of the southern basins in the breeding season might cause thermoregulatory stress for many breeding species. | No | Yes | No |
| 20 | United States (9 million km2) | ? (Only breeding birds and Neotropical migrants) | No | Energy availability at the continental level explained less variance for permanent residents than for neo-tropical migrant species. | No | Yes | No |
| 21 | Europe (10 million km2) | 324 species (only breeding birds) | Yes (impact of Climate Change on long-distant migrants) | The number of migratory species increased towards eastern Europe, and proportion of migratory species increasing towards northeast Europe. Temperature of the coldest month and spring precipitation had a negative effect on number and proportion; and spring temperature had a positive effect on both. | No | Yes* | Yes* |
| 22 | North America (20 million km2) | ? (only breeding birds) | Yes (breeding distribution of migratory birds) | Neo-tropical migrants were less prevalent in grasslands than in forests, but no significant difference in their proportion in deciduous and coniferous forests. Most neo-tropical migrants bred primarily in deciduous forests. | No | No | Yes |
| 23 | Sub-Saharan Africa (20 million km2) | 60 species (only passerine Palearctic migrants) | Yes (determinants of migratory species richness) | Richness in wintering migrants (based on potential distributions) generally decreases with distance from the Sahara Desert. | No | Yes* | No |

^a^ Article describes the pattern of difference in species richness between Northern Hemisphere summer and winter

^b^ Article describes the pattern of richness in migratory species

^c^ Article describes the pattern of proportion of migratory species

* Article describes the pattern spatially with a map

References

^1^ Barcena S, Real R, Olivero J, Vargas JM (2004) Latitudinal trends in breeding waterbird species richness in Europe and their environmental correlates. Biodiversity and Conservation 13: 1997–2014.

^2^ Bohning-Gaese K, Lemoine N (2004) Importance of Climate Change for the ranges, communities and conservation of birds. Advances in Ecological Research 35: 211–236.

^3^ Carnicer J & Diaz-Delgado R (2008) Geographic differences between functional groups in patterns of bird species richness in North America. Acta Oecologica 33: 253–264.

^4^ Chesser RT (1998) Further perspectives on the breeding distribution of migratory birds: South American austral migrant flycatchers. Journal of Animal Ecology 67: 69–77.

^5^ Fuller RJ, Crick HQP (1992) Broad-scale patterns in geographical and habitat distribution of migrant and resident passerines in Britain and Ireland. IBIS 134(1): 14-20.

^6^ Greenberg R, Kozlenko A, Etterson M, Dietsch T (2008) Patterns of density, diversity, and the distribution of migratory strategies in the Russian boreal forest avifauna. Journal of Biogeography 35: 2049–2060.

^7^ Helle P, Fuller RJ (1988) Migrant passerine birds in European forest successions in relation to vegetation height and geographical position. Journal of Animal Ecology 57: 565–579.

^8^ Herrera CM (1978) On the breeding distribution pattern of European migrant birds: Macarthur’s theme reexamined. The Auk 95: 496–509.

^9^ Hockey PAR (2000) Patterns and correlates of bird migration in Sub-Saharan Africa. EMU 100: 401–417.

^10^ Hurlbert AH, Haskell JP (2003) The effect of energy and seasonality on avian species richness and community composition. The American Naturalist 161: 83–97.

^11^ Lemoine N, Bohning-Gaese K (2003) Potential impact of global climate change on species richness of long-distance migrants. Conservation Biology 17: 577–586.

^12^ Lennon JJ, Greenwood JJD, Turner JRG (2000) Bird diversity and environmental gradients in Britain: a test of the species-energy hypothesis. Journal of Animal Ecology 69: 581–598.

^13^ MacArthur RH (1959) On the breeding distribution pattern of North American migrant birds. The Auk 76(3): 318–325.

^14^  Monkkonen M, Forsman JT (2005) Ecological and biogeographical aspects of the distribution of migrants versus residents in European and North American forest bird communities. In: Greenberg R, Marra PP, editors. Baltimore: The Johns Hopkins University Press. pp. 131–142.

^15^ Newton I (1995) Relationship between breeding and wintering ranges in Palaearctic-African migrants. Ibis 137: 241–249.

^16^ Newton I, Dale LC (1996) Relationship between migration and latitude among west European birds. Journal of Animal Ecology 65: 137–146.

^17^ Newton I, Dale LC (1996) Bird migration at different latitudes in Eastern North America. The Auk 113: 626–635.

^18^ Newton I, Dale LC (1997) Effects of seasonal migration on the latitudinal distribution of west Palaearctic bird species. Journal of Biogeography 24: 781–789.

^19^ Olivero J, Real R, Vargas JM (1998) Distribution of breeding, wintering, and resident waterbirds in Europe: biotic regions and the macroclimate. Ornis Fennica 75: 153–175.

^20^ Rowhani P et al. (2008) Variability in energy influences avian distribution patterns across the USA. Ecosystems 11: 854–867.

^21^ Schaefer H, Jetz W, Bohning-Gaese K (2008) Impact of climate change on migratory birds: community reassembly versus adaptation. Global Ecology and Biogeography 17: 38–49.

^22^ Willson MF (1976) The breeding distribution of North American migrant birds: a critique of MacArthur (1959). The Wilson Bulletin 88: 582–587.

^23^ Wisz MS, Walther BA, Rahbek C (2007) Using potential distributions to explore determinants of Western Palaearctic migratory songbird species richness in sub-Saharan Africa. Journal of Biogeography 34: 828–841.
