# Supplementary material for: Mapping Global Diversity Patterns for Migratory Birds
Source: PLoS One. 2013 Aug 7;8(8):e70907. doi: 10.1371/journal.pone.0070907 (PMC3737225; doi:10.1371/journal.pone.0070907)
Supplement: Appendix S2 — (DOC) [file pone.0070907.s002.doc]

**Analysis across Flyways**

A flyway is the total geographic area used by a group of species throughout its annual cycle. The three major global flyways were mapped following the definition of BirdLife International (see <http://www.birdlife.org/flyways/> and Figure S1). Figure S2 shows migratory species diversity as a function of latitude, equivalent of Figure 4 but with different colours for each flyway. To create this figure, we estimated the three major global flyways as follows: the Americas Flyway is defined as continental land whose longitude is west of 30°W, the African-Eurasian Flyway is defined as continental land whose longitude is between 30°W and 60°E, and the East Asian-Australasian Flyway is defined as continental land whose longitude is east 60°E.

**Figure S1**. Three major global flyways (from BirdLife International - <http://www.birdlife.org/flyways/>)


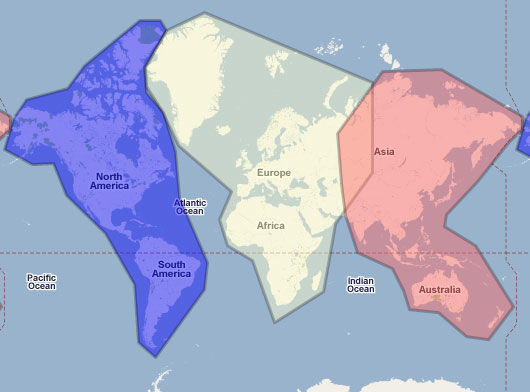


**Figure S2**. Migratory species diversity as a function of latitude across flyways.

(A) Difference in local species richness between July and January; (B) richness in migratory (non-permanent) species; and (C) proportion of migratory (non-permanent) species. The Americas Flyway is in blue, the African-Eurasian Flyway is in green, and the East Asian-Australasian Flyway in red.
