# Supplementary figures and images for: Mapping Global Diversity Patterns for Migratory Birds
Source: PLoS One. 2013 Aug 7;8(8):e70907. doi: 10.1371/journal.pone.0070907 (PMC3737225; doi:10.1371/journal.pone.0070907)

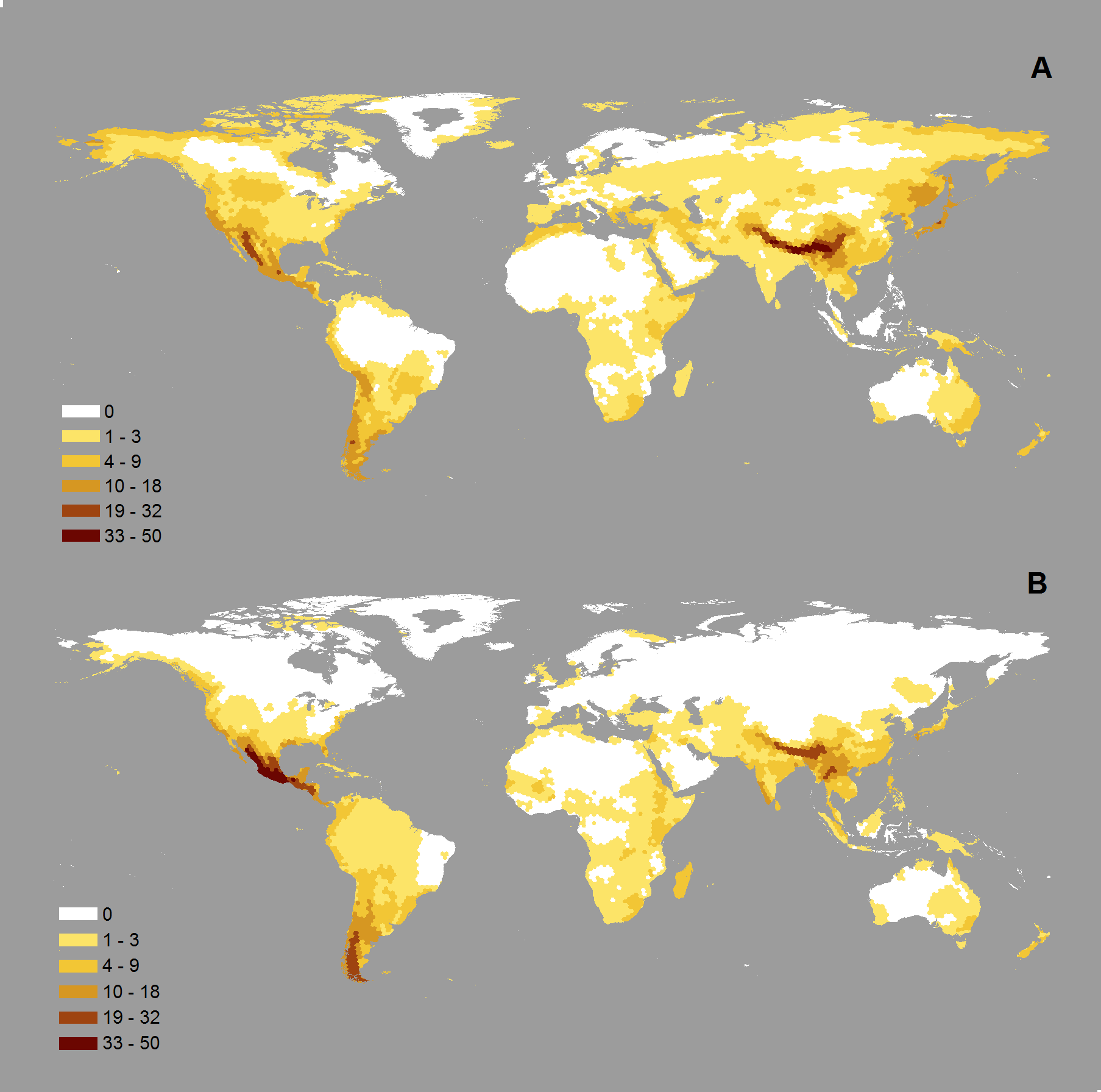

Supplement: File S1 — (TIFF) [file pone.0070907.s003.tiff]
